# Hepatopancreas Transcriptome Profiling Analysis Reveals Physiological Responses to Acute Hypoxia and Reoxygenation in Juvenile Qingtian Paddy Field Carp *Cyprinus carpio var qingtianensis*

**DOI:** 10.3389/fphys.2020.01110

**Published:** 2020-09-11

**Authors:** Ming Qi, Qianqian Wu, Tao Liu, Yiling Hou, Yixin Miao, Menghong Hu, Qigen Liu

**Affiliations:** ^1^Centre for Research on Environmental Ecology and Fish Nutrition of the Ministry of Agriculture, Shanghai Ocean University, Shanghai, China; ^2^Key Laboratory of Freshwater Aquatic Genetic Resources, Ministry of Agriculture, Shanghai Ocean University, Shanghai, China; ^3^Graduate School of Human Development and Environment, Kobe University, Kobe, Japan

**Keywords:** *Cyprinus carpio var qingtianensis*, transcriptome, hepatopancreas, hypoxia, reoxygenation

## Abstract

The Qingtian paddy field carp (*Cyprinus carpio var qingtianensis*) is a local carp cultivated in the rice field of Qingtian county, Zhejiang province, China. Its high tolerance to hypoxia makes it an ideal organism for studying the molecular regulation mechanism during hypoxia process as well as reoxygenation following hypoxia in fish. In this study, we counted the differentially expressed genes (DEGs) altered during hypoxic exposure and reoxygenation process. The results indicated that 2236 genes (1506 up-regulated genes and 730 down-regulated genes) were differentially expressed between the control and hypoxic groups. The results from Kyoto Encyclopedia of Genes and Genomes (KEGG) enrichment analysis indicated that 1152 of 2236 genes were enriched, and those genes participated in energy metabolism, reactive oxygen species (ROS) elimination, acceleration of cell apoptosis, inhibition of growth, and other processes. We found activation of the pentose phosphate pathway in hypoxia treatment, suggesting that carbohydrates not only provide energy for metabolism but also provide NADPH for protecting the body from oxidative damage and ribosomes for promoting RNA synthesis. During reoxygenation, 4509 genes (1865 up-regulated genes and 2644 down-regulated genes) were differentially expressed. The results of KEGG enrichment analysis indicated that 2392 of 4509 genes were enriched, and participated in pyruvate and lactic acid metabolism, synthesis of amino acids and lipids, inhibition of cell apoptosis, regulation of cell growth and differentiation, and other processes. These differentially expressed genes effectively alleviate the body acidosis and promote the normal growth and development of the body. Through the analysis of KEGG pathway enrichment, we observed that the physiological regulation of Qingtian paddy field carp during the processes of hypoxia and reoxygenation is not a simple and reversible process. This work first reported the adaptive mechanism of hypoxia and the recovery mechanism of reoxygenation after hypoxia in common carp, and also provided new insights for the physiological regulation of fish under hypoxia treatment.

## Introduction

Fish are prone to stress in response to low dissolved oxygen (DO) levels, which is extremely common in aquatic ecosystems due to natural causes such as diurnal oscillations in algal respiration, seasonal flooding, stratification, and isolation of densely vegetated water bodies, as well as to more recent anthropogenic causes (e.g., eutrophication) (Hughes, 1973; Herbert and Steffensen, 2005; Gooday et al., 2009). It is even more common for fish to encounter hypoxic stress in aquaculture systems where fish are often cultured with high densities or are limited by the environmental conditions and farming modes such as in the shallow rice fields (e.g., rice-fish co-culture). In response to long-term hypoxic stress, fish might solve these problems by avoidance, or by adaptive evolution (e.g., specialized respiratory organs and increased blood vessels in skin) (Mandic et al., 2008; Shields and Knight, 2011; Zaccone et al., 2018). Stress response to acute hypoxia can be easily induced in fish. Moreover, fish are especially vulnerable to oxidative damage from rapid reoxygenation following hypoxic stress (Hermes-Lima and Zenteno-Savin, 2002). They reduce damage caused by hypoxia and acute reoxygenation through their physiological metabolism regulation by increasing antioxidant enzyme activities, O_2_ affinity of blood, and by reducing energy available for biosynthesis (e.g., synthesis of glycogen and lipid) (Hughes, 1973; Ishimatsu and Itazawa, 1983; Cooper et al., 2002; Wu, 2009; Wang and Richards, 2011). The damage of hypoxia to fish includes reduced growth and reproduction and death in severe cases (Pérès et al., 1973; Wu, 2009; Shields and Knight, 2011). Fish can adapt to hypoxia to reduce the possible damage by decreasing basal metabolism and increasing energy metabolism (Muusze et al., 1998). When fish are subjected to hypoxia in aquaculture, people usually resort to increase dissolved oxygen in the water to alleviate the hypoxic conditions. However, due to the lack of systematic studies, it is yet unknown whether the reoxygenation of fish following hypoxia is a simple reversible physiological process.

To understand the physiological responses in the reoxygenation of fish after hypoxia, previous studies primarily analyzed the changes of antioxidant enzyme activity, energy metabolism, and other phenotypic physiological changes (Hughes, 1973; Thomas et al., 1988; Muusze et al., 1998). In addition, some researchers have studied the regulatory mechanism of hypoxic adaptability in aquatic animals through transcriptome, proteome, metabolome, and other molecular biological techniques. However, no efforts have been made to explore the whole physiological response and regulatory mechanism of reoxygenation following hypoxia. In this study, the Qingtian paddy field carp (PF-carp), also known as the Oujiang color carp (Wang et al., 2006; Xie et al., 2011) (*Cyprinus carpio var. color*) was chosen as a typical model. These fish have been co-cultured with rice in paddy fields for more than 1200 years as they showed strong tolerance to hypoxia (Ren et al., 2018). At present, the research on PF-carp mainly focused on the symbiotic relationship between rice and fish, body color, et.al (Wang et al., 2006; Xie et al., 2011; Ren et al., 2018). The dissolved oxygen concentration in this rice-fishery symbiotic system has been reported to be higher than 6 mg/L, and there is a significant diurnal variation in dissolved oxygen (Guo et al., 2020). PF-carp was used in this study because this species had already become well adapted to shallow rice fields during the long history of co-culturing of rice and fish and they may have evolved the capability of strong tolerance to hypoxia. However, the mechanism of hypoxia adaptation has not been clarified yet, thus it is essential to explore its physiological response and molecular regulatory mechanisms to hypoxia and reoxygenation.

RNA-seq had been successfully applied to the study of environmental toxicology, nutritional intervention and growth and development of fish (Wang et al., 2016; Caballero-Solares et al., 2018; Qi et al., 2018). RNA-seq analysis is an appropriate analysis for this study, because it can effectively differentiate differentially expressed genes (DEGs) between different test groups. We also used RNA-seq to screen DEGs between the control and the hypoxic and reoxygenated groups as well as Kyoto Encyclopedia of Genes and Genomes (KEGG) pathway enrichment analysis to understand high-level functions and utilities of the hepatopancreas in response to hypoxia and reoxygenation. Our main objectives were to determine the acting genes that are involved in physiological response to hypoxia and the molecular mechanisms for this carp to reoxygenation following acute hypoxia.

## Materials and Methods

### Experimental Fish, Acute Hypoxia and Reoxygenation Exposure Experiment

Healthy juvenile PF-carps were transferred from the Yugong ecological agriculture technology Co, Ltd (Qingtian, Zhejiang, China) to the fisheries ecology laboratory at Shanghai Ocean University. Three polyethylene tanks (250-L) were prepared and 9 PF-carps were randomly kept in each tank for 2 weeks prior to the acute hypoxic and reoxygenation experiments. Their individual specifications are 56.64 ± 10.74 g in weight, 15.36 ± 1.29 cm in length, and 102 days old. The fish were fed with artificial feed (1% of the weight of fish: 30% crude protein content and 3% crude fat content; Techbank, China) at 8:00 and water was changed (50% of the volume of in the tank) at 18:00 once a day for two weeks before the experiments. Water temperature was maintained at 25.17 ± 0.41°C and DO was controlled at 6.56 ± 0.20 mg/L. Feeding was stopped and all of the water was replaced one day before the experiment was conducted; multifunctional dissolved oxygen instrument (YSIPro20, United States) was used to detect water temperature and dissolved oxygen every 10 min. In order to reduce the stress resulted from changing water on fish, the juvenile carps were allowed to acclimated for 6 h before the experiment.

At the beginning of the experiment, 3 juvenile PF-carps were picked from each of the three tanks and euthanized with a concentration of 0.3 mg/L MS-222. Their hepatopancreases were excised and immediately frozen in liquid nitrogen. The samples were transferred to a −80°C freezer. Total RNA was subsequently extracted from mixtures of samples from each group of three fish. The DO during this process was 6.53 ± 0.41 mg/L (hereafter, control group as CH). Next, we quickly flushed the water of the three tanks with N_2_ to reduce the level of dissolved oxygen until the DO was at about 0.5 mg/L, when the juvenile carps swam to surface because of hypoxia. The hypoxia treatment was regulated by flows of N_2_ and O_2_ to maintain DO concentration of 0.5 mg/L with water temperature of 25°C. The hypoxic stress experiment lasted for 6 h, during which DO was averaged at 0.53 ± 0.07 mg/L and temperature averaged 25.37 ± 0.45°C. Samples were collected at 6 h in the same way as the samples taken from the CH group (hereafter, hypoxic stress group as HH). The RNA-seq results were defined as HH. At the end of the hypoxic stress experiment, injection of N_2_ into the water was stopped and O_2_ was rapidly injected to raise the level of dissolved oxygen in the water of the three tanks until they reached about 7 mg/L. Then the injection of O_2_ was regulated to maintain a constant high level. The reoxygenation process lasted for 6 h and the DO was averaged 6.64 ± 0.18 mg/L and temperatures averaged 25.21 ± 0.37°C (hereafter, reoxygenation group as RH). Samples were collected after 6 h of reoxygenation in the same way as samples from the CH group. The results of RNA-seq were defined as RH.

### RNA Extraction, Library Preparation, and Sequencing

Total RNAs of hepatopancreas (*n* = 3 per group) was isolated using the TRIzol (Invitrogen, Carlsbad, CA, United States) reagent following the manufacturer’s instructions, and genomic DNA was removed with DNase I (Takara, Shanghai, China). Then, RNA quality was determined by Bioanalyzer 2100 (Agilent Technologies, United States) and quantified using the ND-2000 (NanoDrop Technologies, United States). Only high-quality RNA samples (OD 260/280 ≥ 1.9, OD 260/230 ≥ 1.5, RIN ≥ 8.0) were used to construct a sequencing library.

After quality control, these RNAs were reversed into cDNAs for sequencing as 150 bp paired-end reads on Illumina NovaSeq 6000 (Illumina, United States) by Major Bioinformatics Technology Co., Ltd (Shanghai, China).

### Quality Control and Sequence Alignment

The image signals of sequences were output by Illumina Novaseq 6000 sequencing system, and converted into text data after CASAVA base calling and stored in FASTQ format as raw data (raw reads). After quality control, clean reads were obtained by using SeqPrep and Sickle software (Hirsch et al., 2014) to remove the splice and N (N: modular base) sequences from raw reads and to filter the low quality sequences (quality value < 20). After quality control, the error rate, Q20, Q30, and GC contents of clean reads were calculated to evaluate the sequencing quality.

Based on the improved BWT (Burrows - Wheeler transform) algorithm, clean reads were compared with the common carp genome (*Cyprinus carpio*, GCF_000951615.1, https://www.ncbi.nlm.nih.gov/assembly/GCF_000951615.1/) (Xu et al., 2014) using HISAT v.2 2.1.0 software (Kim et al., 2019) to obtain mapped reads for subsequent analysis.

All data sets of transcriptome had been submitted to the National Center for Biotechnology Information (NCBI) Sequence Read Archive (SRA) database (accession number SRX7829257).

### Transcriptome Assembly and Annotation

With the genome information of carp as reference, the mapped reads were spliced and finally assembled into transcripts using Cufflinks v. 2.2.1 software (Trapnell et al., 2012). Then GffCompare was used to compare the assembled transcript with the known transcript, and unknown genes and new transcripts were identified. The transcriptomes assembled were annotated using BLAST + v.2.9.0 against the NCBI non-redundant protein database (Nr represent as follow), the Swiss-Port database (Swiss-Port), the Clusters of Orthologous Groups of protein database (COG), the GO Ontology database (GO), the Kyoto Encyclopedia of Genes and Genomes database (KEGG) and the Pfam protein families database (Pfam) and annotation information was procured.

### Differential Expression Analysis of Genes and Enrichment Analysis

Gene expression values were calculated using the TPM (transcripts per million reads) value in RESM v.1.3.1 software. For libraries of three experimental groups, the read counts were adjusted by DEGseq2 v. 1.24.0 (Robinson et al., 2010) through one scaling normalized factor. Wilcoxon rank sun test was used to correct *P*-values to obtain adjusted *P*-value (*P*-adjust) during the statistical calculation of differential expressions. And then *P*-adjusted < 0.05 and | log2(foldchange)| ≥ 1 were set as the threshold for significantly differences to screen for the differentially expressed genes (DEGs).

The Goatools v. 0.6.5 software (Klopfenstein et al., 2018) was used for GO enrichment analysis of the DEGs, so as to obtain the main GO functions of DEGs. The DEGs were analyzed by KOBAS v.2.1 (Mao et al., 2005)and the result of the KEGG enrichment obtained. When *P*-adjusted < 0.05, GO functions and KEGG pathways were considered to be significantly enriched. In order to understand the changes of physiological metabolism of PF-carp in the process of hypoxia and reoxygenation, KEGG pathway classifications of Metabolism, Cellular Processes, Environmental Information, Organismal Systems and Genetic Information Processing were screened for subsequent analysis.

### Real-Time Quantitative PCR Validation

In order to evaluate the accuracy of transcriptome sequencing results, eleven DEGs that differentially expressed more than 2 times were randomly selected from the CH, HH and RH groups for RT-qPCR verification. Primers for DEGs were designed in Primer Premier v.6.0 for RT-qPCRs based on mRNA sequences of the common carp. To standardize the gene expression levels of the DEGs in different groups, *apr* (acidic ribosomal phosphoprotein P0) and β*-actin* (beta-actin) were selected as internal reference genes. According to the experimental requirements of the real-time fluorescence quantitative PCR instrument (ABI 7500, United States), 20 μL of a solution [16.5 μL ChamQ SYBR Color qPCR Master Mix(2x), 0.8 μL of each primer (5 μM), and 2 μL template (cDNA)] was used. The PCR reaction conditions were as follows: 95°C for 5 min, followed by 40 cycles of 95°C for 5 s, 50 or 55°C for 30 s and 72°C for 40 s. By comparing CT(ΔΔCT) values, the relative expression was estimated between different groups. Three biological replicates and three technical replicates were performed on the RT-qPCR experiment of DEGs.

GraphPad Prism 7.0 software was used to analyze the results of gene differential expression in RT-qPCR and RNA-seq, and the results were displayed with graphs.

## Results

### Quality Assessment of Transcriptome Sequencing Data

9 separate cDNA libraries from three groups were sequenced by Illumina Novaseq 6000 paired-end sequencing system to produce 66.86 Gb of raw bases. We obtained 440,641,918 raw reads (66.86 Gb), of which 437,430,924 passed the quality control processes. The data size of clean reads per library ranged from 6.54 Gb∼7.73 Gb. At the same time, the clean reads were compared with the reference genome. Parameter statistics of clean reads among the control, hypoxia and reoxygenated groups were Q20: 98.33∼98.89%; Q30: 94.63∼96.35%; GC content: 48.15∼49.12%; error rate: 0.023∼0.024%. Clean reads of each library performed sequence alignment with the reference genome information of common carp to obtain mapped reads, and total ratio of each sample was 79.29%∼80.91% (Table 1).

**TABLE 1:** Statistical of the raw and clean sequencing of the RNA-seq library of *Cyprinus carpio var qingtianensis.*

Sample	Raw reads	Clean reads	Clean bases (Gb)	Q20 (%)	Q30 (%)	GC content (%)	Error rate (%)	Total mapped rate (%)
**Control (Normoxia) Groups**								
CH1	47786542	47435484	7.12	98.86	96.24	49.12	0.023	79.83
CH2	50685024	50314602	7.54	98.89	96.35	48.95	0.023	80.84
CH3	49671818	49279064	7.37	98.78	96.04	48.19	0.023	79.29
**Hypoxia Groups**								
HH1	50458850	50083904	7.50	98.79	96.08	49.03	0.023	79.93
HH2	48358862	48004638	7.19	98.83	96.19	48.73	0.023	80.51
HH3	43873942	43591086	6.54	98.33	94.63	48.49	0.024	80.68
**Reoxygenated Groups**								
RH1	47899832	47557022	7.12	98.81	96.14	48.15	0.023	80.37
RH2	49889836	49535028	7.42	98.83	96.18	48.63	0.023	79.52
RH3	52017212	51630096	7.73	98.83	96.17	48.49	0.023	80.91

*CH, HH, and RH represent hepatopancreas of *Cyprinus carpio var qingtianensis* were treated under normoxic, hypoxic and reoxygenated conditions for 6 h.*

### Transcriptome Assembly and Annotation

**FIGURE 1:**
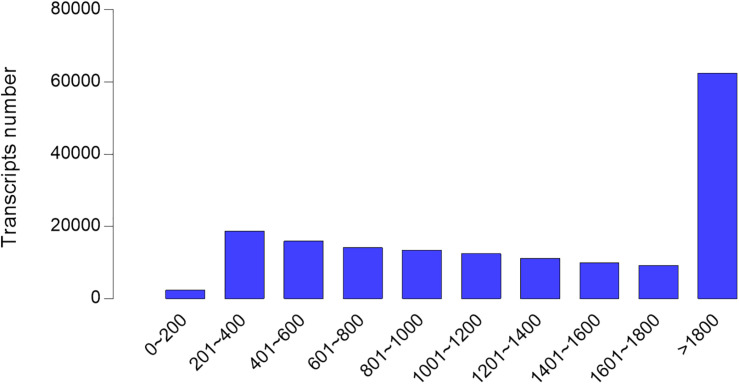
Transcript length distribution after assembled in hepatopancreases of *Cyprinus carpio var qingtianensis.* Most of the assembled transcripts are long transcripts, indicating that the quality of the assembled transcripts is good.

**FIGURE 2:**
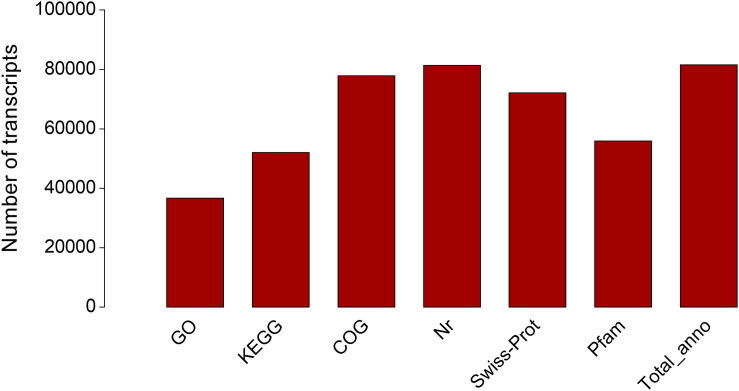
Transcript annotation information in hepatopancreases of *Cyprinus carpio var qingtianensis.*

According to the information of mapped reads, a total of 86,524 transcripts were assembled by Cufflinks (Figure 1). The assembled transcripts were annotated against six public databases (NR, Swiss-Prot, COG, GO, KEGG, Pfam) with 81,408, 72,157, 77,905, 36,715,52,093, and 5,5959 transcripts, respectively, and 81,534 transcripts can be annotated effectively in six databases (Figure 2).

### Differential Genes Expression Analysis

**FIGURE 3:**
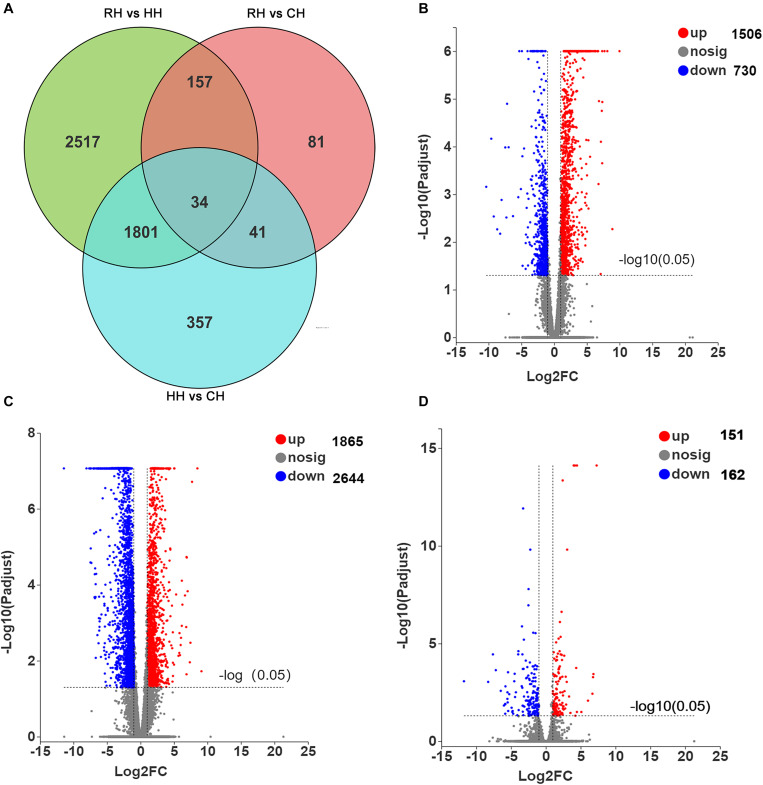
Statistics of DEGs in HH vs. CH, RH vs. HH, and RH vs. CH groups, **(A)** Venn diagram of DEGs in HH vs. CH, RH vs. HH, and RH vs. CH groups, **(B)** Volcano plot of DEGs in HH vs. CH group, **(C)** Volcano plot of DEGs in RH vs. HH group, and **(D)** Volcano plot of DEGs in RH vs. CH group.

In this study, *P-*adjust < 0.05 and| log2(foldchange)| ≥ 1 were used to identify significant DEGs. The results showed that a total of 4988 DEGs among CH, HH, and RH (Figure 3A), including 2236 DEGs (1506 up-regulated and 730 down-regulated) between the HH and CH (Figure 3B), 4509 DEGs (1865 up-regulated and 2644 down-regulated) between the RH and HH (Figure 3C), 313 DEGs (151 up-regulated and 162 down-regulated) between the RH and CH (Figure 3D).

**FIGURE 4:**
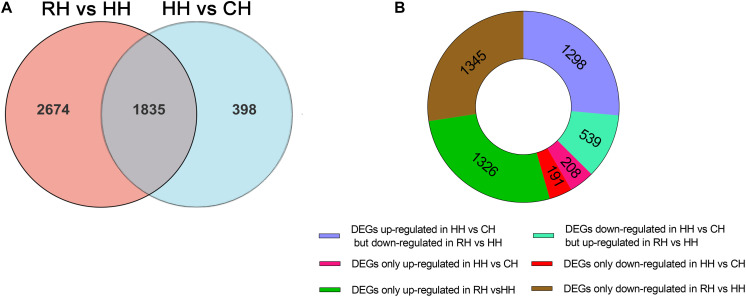
Statistics of differentially expressed genes in RH vs. HH and HH vs. CH groups; **(A)** Venn figure of DEGs; **(B)** Ring chart of expression patterns of DEGs.

The expression patterns of the DEGs (4907) in between HH versus CH and RH versus HH groups were divided into 8 groups (Figure 4A): (1) up-regulated in HH versus CH but down-regulated in RH versus HH (1298), (2) down-regulated in RH versus HH but up-regulated in HH versus CH (539), (3) both up-regulated in HH versus CH and RH versus HH (0), (4) both down-regulated in HH versus CH and RH versus HH(0), (5) up-regulated in HH versus CH only (208), (6) down-regulated in HH versus CH only (191), (7) up-regulated in RH versus HH only(1326), and (8) down-regulated in RH versus HH only (1345) (Figure 4B).

**FIGURE 5:**
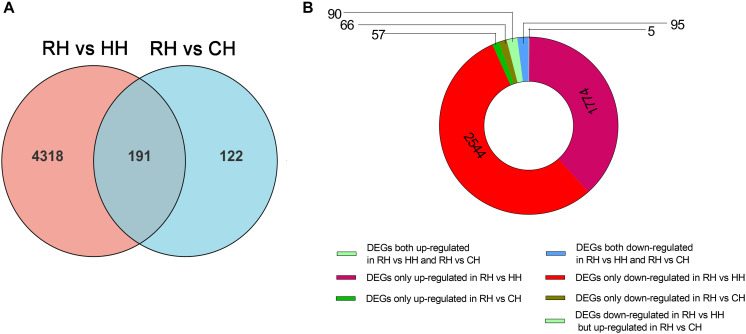
Statistics of differentially expressed genes in RH vs. HH and RH vs. CH groups, **(A)** Venn figure of DEGs, **(B)** Ring chart of expression patterns of DEGs.

At the same time, in order to evaluate the reoxygenation recovery status of PF-carps, the comparison of DEGs in RH vs. CH groups revealed that there were only 313 DEGs, suggesting that the physiological state of PF-carps basically recovered after 6 h of reoxygenation following hypoxia. However, in order to better explain the physiological regulation mechanism of PF-carp after reoxygenation, the DEGs (4631) of RH versus HH and RH versus CH groups were compared and divided into 8 groups (Figure 5A): (1) up-regulated in RH versus HH but down-regulated in RH versus CH groups (0), (2) down-regulated in RH versus HH groups but up-regulated in RH versus CH groups (5), (3) both up-regulated in RH versus HH groups and RH versus CH (90), (4) both down-regulated in RH versus HH groups and RH versus CH(95), (5) up-regulated in RH versus HH groups only (1774), (6) down-regulated in RH versus HH groups only (2544), (7) up-regulated in RH versus CH only (57), and (8) down-regulated in RH versus CH only (66) (Figure 5B).

### GO Function Enrichment Analysis of DEGs

**FIGURE 6:**
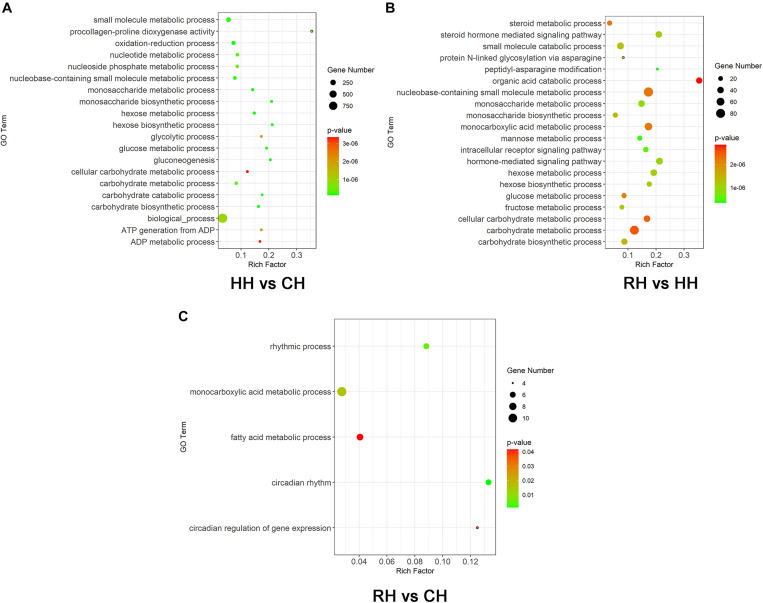
GO function enrichment analysis of DEGs, **(A)** HH vs. CH group (the top 20 most enriched term), **(B)** RH vs. HH group (the top 20 most enriched term), and **(C)** RH vs. CH. The color of each dot indicated the corrected *P*-adjust for the corresponding terms.

The all DEGs in each treatment and comparison group were screened for GO functional enrichment analysis and classified into biological process (BP), cellular component (CC) and molecular function (MF). Compared to normoxic (control) group, 785 of 1506 up-regulated DEGs of hypoxic group were significantly enriched in 222 GO terms, including 159 biological process, 10 cellular component, 53 molecular function and 367 of 730 down-regulated DEGs of hypoxia group were significantly enriched in 56 GO terms, including 40 biological process, 6 cellular component, 10 molecular function, the GO terms mainly involved oxidation-reduction, nucleotide metabolic process and glucose metabolic process (Figure 6A for the top 20 most enriched term). 1044 of 11865 up-regulated DEGs of RH versus HH group were significantly enriched in 118 GO terms, including 73 biological process, 17 cellular component, 28 molecular function and 1348 of 2644 down-regulated DEGs of RH versus HH group were significantly enriched in 319 GO terms, including 218 biological process, 13 cellular component, 87 molecular function, the GO terms mainly involved steroid metabolic process, monosaccharide biosynthetic process and carbohydrate biosynthetic process (Figure 6B for the top 20 most enriched term). 95 of 151 up-regulated DEGs were significantly enriched to 10 GO terms in the reoxygenated group. Compared with that in the normoxic (control) group, including 8 biological process, 2 cellular component and 77 of 162 down-regulated DEGs were significantly enriched to only one cellular component, the GO terms mainly involved monocarboxylic acid metabolic process, fatty acid metabolic process and circadian regulation of gene expression (Figure 6C).

### KEGG Pathway Enrichment Analysis of DEGs

The results showed 1580 DEGs of the KEGG pathway were significantly enriched in the HH versus CH comparison, 3222 DEGs were significantly enriched in the RH versus HH comparison and 224 DEGs were significantly enriched in the RH versus CH comparison.

**FIGURE 7:**
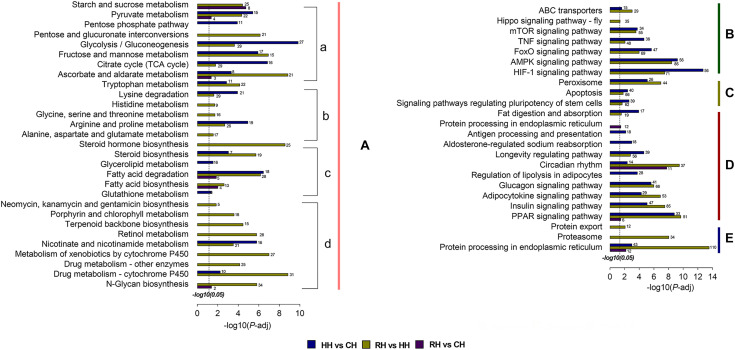
KEGG pathway enrichment analysis of DEGs in HH vs. CH, RH vs. HH, and RH vs. CH. **(A)** Metabolism: **(a)** Carbohydrate metabolism, **(b)** Amino acid metabolism, **(c)** Lipid metabolism, and **(d)** Others metabolism; **(B)** Environmental information processing; **(C)** Cellular processes; **(D)** Organismal systems; **(E)** Genetic information processing. The length of each column indicated the -log10(*P*-adjust) of the corresponding pathway. The annotation number indicated the number of DEGs were enriched in the corresponding pathways.

There were total of 35 KEGG pathways enriched by DEGs in the HH versus CH group, and the pathways with a relatively large number of enriched genes were HIF-1 signaling pathway (56 genes), glycolysis/gluconeogenesis (27 genes), pentose phosphate pathway (11 genes). Among the 48 KEGG pathways enriched by the DEGs in the RH versus HH group, the DEGs were mainly enriched in HIF-1 signaling pathway (71 genes), pentose and glucuronate interconversions (21 genes), steroid hormone biosynthesis (25 genes). However, the DEGs in the RH versus CH group enriched to 11 signaling pathways, mainly protein processing in endoplasmic reticulum (12 genes), fatty acid biosynthesis (4 genes), pyruvate metabolism (4 genes) (Figure 7).

### The Verification of RNA-seq by RT-qPCR

**FIGURE 8:**
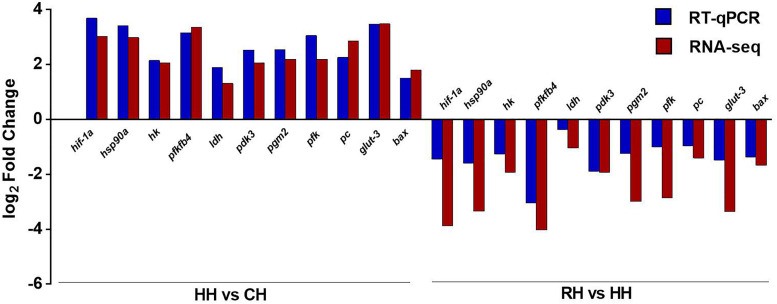
Comparison of gene expression between RT-qPCR and RNA-seq. The *X*-axis represents the gene name, and the *Y*-axis represents the log_2_ value of the relative gene expression between the two groups. The length of the blue column represents the average of log_2_ value of the relative gene expression between the two groups by RT-qPCR, the length of the red column represents the average of log_2_ value of the relative gene expression between the two groups by RNA-seq.

The RT-qPCR was performed with designed primers to obtain the expression results of DEGs (Table 2). The results showed that RT-qPCR of 11 DEGs were consistent with RNA-seq in HH versus CH group. In the RH versus HH group, the relative expression levels of *hif-1a*, *hsp90a*, *ldh*, *pgm2*, *pfk*, and *glut-3* genes in RT-qPCR were different from that of RNA-seq, but their up-down expression trends were consistent. The accuracy of RNA-seq was verified (Figure 8).

**TABLE 2:** Primer sequences for RT-qPCR verification.

Primer (Gene’s name)	Gene ID	Forward primers Sequence (5′-3′)	Reverse primers Sequence (5′-3′)
hif-1a	XM_019117116.1	AGGCACTGGCAGGCTTTA	GTGAGGATGTCTCGCAAT
hsp90a	XM_019071000.1	CAGAAGCCGACAAGAATG	TGTAGATGCGGTTGGAGT
hk	XM_019104220.1	AAGAGCAAAGAGGGACTTA	CCAGAGTAGCAGCAATCA
pfkfb4	XM_019084601.1	CACTACCGCTACCCTAAA	AGCAGACAACGCATAACA
ldh	XM_019112676.1	ATGTGGCAGGAGTCAATCT	CGTGTAGCCTTTCAGTCG
pdk3	XM_019104220.1	GAACAGGAAGCCAGATGA	TTCGGTTGGTGTAGAAGC
pgm2	XM_019116956.1	GATGTTTGAGCGTCTTCG	GCTGTCATAGCCAGTCGTA
pfk	XM_019074709.1	TGCCACAAGCACTACACC	CACAGAACTGACTTCACTCCC
pc	XM_019125172.1	ACACTCGCCTGTTTCTGG	CATTGCTGCCGTATCCTT
glut-3	XM_019090916.1	GACTGCCATCCCTGAATC	TGGACCTTGAGCGAATAA
bax	XM_019093006.1	ACTTTGCGTGTCGGCTTGT	CCTCCCAYCCACCCTGTT
arp	XM_019096904.1	TGGGAATCACCACCAAGA	GCTGCCGTTATCATACACC
β-actin	XM_019089432.1	ACCCTGGCATTGCTGACC	CGTACTCCTGCTTGCTGATC
			

## Discussion

Dissolved oxygen is one of the important environmental factors that limit fish survival (Abdel-Tawwab et al., 2019). In fish, the liver (for PF-carp is hepatopancreas) is the central organ of metabolism and important defensive organ (Guilherme et al., 2012). Changes in dissolved oxygen can affect the physiological function of the liver and cause damage, such as energy metabolism, antioxidant stress and anabolism of other substances (Muusze et al., 1998; Wang and Richards, 2011; Jung et al., 2014). At the same time, how the liver (hepatopancreas) will adapt to the reoxygenation following hypoxia and its molecular regulatory mechanism is not clear. Therefore, it is significance to use RNA-seq to analyze the response mechanism of the liver during hypoxia and reoxygenation.

In this study, we used RNA-seq to screen and compare the DEGs in hepatopancreas of PF-carp during hypoxia and reoxygenation. The results showed that the number of up-regulated DEGs exceeded the number of down-regulated DEGs during hypoxia, which is consistent with the results reported for the hypoxic transcriptome analysis of *Pelteobagrus vachell* (Zhang et al., 2016). In contrast, the number of down-regulated DEGs exceeded the number of up-regulated DEGs during reoxygenation, which suggested that PF-carp had enhanced physiological metabolism against stress during the hypoxic process, while the metabolism was mainly reduced in the reoxygenation process (e.g., cell apoptosis and glycolysis) (Sun et al., 2017). Activation and inhibition of the corresponding metabolic pathways appear to be a reversible physiological regulation, but activation of some metabolic pathways counters this hypothesis. Take carbohydrate metabolism for example, the pentose phosphate pathway was activated only during hypoxia and pentose and glucuronate interconversions was activated only during reoxygenation.

HIF-1a is an important homeostasis regulatory protein in organisms (Baptista et al., 2016). In this study, the *hif-1a* gene was up-regulated during hypoxia and down-regulated following reoxygenation (Figure 7), suggesting that a molecular regulatory pathway with HIF-1 signal pathway as the core occurred in PF-carp during the hypoxic and reoxygenation processes (Zhang et al., 2017b), thereby triggering a series of metabolic changes that includes energy metabolism (Goda and Kanai, 2012), cell proliferation and apoptosis (Carmeliet et al., 1998), immune response (Cramer et al., 2005) and other metabolic pathway (Semenza, 1999).

We observed that DEGs in HH versus CH and RH versus HH groups were significantly enriched for glycolysis/gluconeogenesis, fructose and mannose metabolism, pyruvate metabolism, and TCA cycle. This is similar to previous studies of fish energy metabolism based on carbohydrate during hypoxia and reoxygenation (Li et al., 2018). The expression of *pfk, hk*, and *ldh* genes were up-regulated to enhance the glycolysis and lactic acid metabolism pathway and *acly* genes was down-regulated and to inhibit the TCA cycle, which resulted in the transformation of hepatopancreatic cells from aerobic respiration to anaerobic respiration during hypoxia (Ding et al., 2020). In the process of reoxygenation, *pfk, hk*, and *ldh* genes were down-regulated and the glycolysis pathway was inhibited, while the *acly* genes was up-regulated and TCA cycle was activated, suggesting that the aerobic respiration of the body was restored, and the substrate for energy metabolism was lactic acid accumulated during hypoxia. This result was verified by using P-NMR to study the changes of muscle energy during hypoxia and reoxygenation (Wright et al., 1989; van Ginneken et al., 1995). At the same time, the catabolism of lactic acid can effectively reduce acidosis in hepatopancreas (Fiévet et al., 1987). Interestingly, we found that the DEGs were significantly enriched in the pentose phosphate pathway during hypoxia. The pentose phosphate pathway is activated during hypoxia because the pentose phosphate pathway, on the one hand, provides the metabolic substrate for carbohydrate metabolism, and on the other hand, most of the NADPH and ribose in the body are produced through the pentose phosphate pathway (Hu et al., 2012). NADPH is a reducing agent in the body, which effectively protects glutathione activity and removes reactive oxygen species (ROS) from the body (Mailloux et al., 2013; Jin et al., 2018). Ribose provides more raw materials for the synthesis of RNA, ensuring that up-regulated gene transcribed into RNA (Cleaves and Miller, 2001). With regards to amino acid and lipid metabolism, on the one hand, amino acid and lipid metabolism are activated to provide adequate substrate for energy metabolism (Wright et al., 1989) and reduced synthesis of amino acids and lipids under hypoxia (Zhang et al., 2017a). On the other hand, the energy metabolism of substrates for lactic acid and pyruvic acid are reduced during reoxygenation. Moreover, high carbohydrate levels result in synthesis of amino acids and lipids (Li and Zhang, 2016). *g6pd* and *gshB* genes expressions were up-regulated in the process of reoxygenation, which accelerated glutathione biosynthesis to reduce oxidative damage of hepatopancreas cells (Lu, 2013). *agl5* gene is the key gene to regulate the synthesis of N-glycan, and its expression is continuously up-regulated after reoxygenation, which will promote the synthesis of N-glycan. This may be related to the promotion of glycosylation of membrane proteins and repair of damaged cell membranes (Sprovieri and Martino, 2018).

When fish are under hypoxia stress, extracellular signaling factors bind specifically to intracellular or cell membrane receptors to induce intracellular signaling, triggering a cascade reaction thereby affecting cell function. The signal transduction in the cell is a variety of interrelated signal transduction patterns (Pugh and Ratcliffe, 2017). For example, the HIF-1 signaling pathway and the Toll signaling pathway coordinate through the activation travel of TLR4 (toll-like receptor) and NF-kB (nuclear factor), inducing the generation of inflammation (Rius et al., 2008; Wu et al., 2018). Zebrafish embryos regulate the maturation and differentiation of intestinal goblet cells through the expression of the *agr2* gene mediated by HIF-1a and Foxa2 (Lai et al., 2016). By regulating AMPK (AMP activated protein kinase) activity during hypoxia, goldfish complete the distribution and recombination of intracellular energy metabolism (Lau and Richards, 2011). In this study, we observed that during the processes of hypoxia and reoxygenation, signal transduction pathways of PF-carp were regulated by the changes of dissolved oxygen (activation/inhibition of HIF-1 signaling pathway), and HIF-1 signaling was regulated by the coordination of Toll, AMPK, FoxO, and TNF pathways to complete cell proliferation, differentiation, immune response, and energy distribution (Lau and Richards, 2011; Lai et al., 2016; Wang et al., 2018; Wu et al., 2018). In this study, PF-carp hepatopancreatic DEGs significantly activated and inhibited the HIF-1 signaling pathway during the processes of hypoxia and reoxygenation, and then coordinated the Toll, AMPK, FoxO, and TNF signaling pathways to complete cell differentiation and maturation, energy redistribution, and metabolic regulation induced by inflammation.

During hypoxia, a large number of ROS were produced in the hepatopancreas of PF-carp. up-regulated expressions of *acox1* and *sod1* activated the peroxidase pathway to remove ROS from the body (Welker et al., 2012). Meanwhile, ROS cause the oxidation of membrane proteins and unsaturated fatty acids and damage DNA. Hepatopancreas cells could enhance the apoptosis pathway and clean up the damaged cells by up-regulating the expression of *bax, caspase8*, and *hsps*, which effectively protect the function of hepatopancreas (Poon et al., 2007). The difference is that the apoptosis pathway is inhibited by down-regulated expression of key genes (i.e., *bcl-2* and *mcl1*) and the peroxisome pathway is continuously activated through up-regulated expression of key genes (i.e., *pex1*, *sod1*, and *acox1*) during reoxygenation. Sustained activation of the peroxisome pathway is due to a new round of oxidative stress by reoxygenation, which is similar to the changes in the oxygen supply of goldfish (Hermes-Lima and Zenteno-Savin, 2002).

## Conclusion

In this study, RNA-seq analysis was used to compare the changes of expression of genes in hepatopancreas in PF-carp during hypoxia and reoxygenation. Compared the number of up-regulated and down-regulated genes between each different treatment group, we found that the adaptive regulation of PF-carp was achieved by activating the metabolic processes during hypoxia and inhibiting metabolism during reoxygenation. At the same time, through the analysis of KEGG pathways enriched by DEGs during hypoxia and reoxygenation, this regulation is not a simple reversible physiological regulation. After 6-h period of reoxygenation following 6 h of hypoxia, PF-carp continued to be up-regulated some genes regulating metabolic pathways, which are very important for cell repair, and the expression of other genes basically returned to the initial level. It is significance to understand the adaptability of PF-carp to the changing environment of dissolved oxygen in rice field. At the same time, gene expression does not mean the execution of functions, which requires us to further improve the study on the adaptability of PF-carp to changes in acute dissolved oxygen through other research methods.

## Data Availability Statement

The datasets presented in this study can be found in online repositories. The names of the repository/repositories and accession number(s) can be found below: https://www.ncbi.nlm.nih.gov/, SRX7829257.

## Ethics Statement

This study was approved by the Institutional Animal Care and Use Committee (IACUS) of Shanghai Ocean University (Shanghai, China).

## Author Contributions

MQ, MH, and QL designed the study and wrote the manuscript. TL, YM, and YH conducted the experiment and collected the materials. MQ, QW, and MH performed the experiments and analyzed the data. All authors have read and approved the final manuscript.

## Conflict of Interest

The authors declare that the research was conducted in the absence of any commercial or financial relationships that could be construed as a potential conflict of interest.
